# A1C predicts type 2 diabetes and impaired glucose tolerance in a population at risk: the community diabetes prevention project

**DOI:** 10.1186/1758-5996-1-5

**Published:** 2009-09-16

**Authors:** Silmara AO Leite, Robyn L Anderson, David M Kendall, Arlene M Monk, Richard M Bergenstal

**Affiliations:** 1 Universidade Positivo, Curitiba, PR, Brazil; 2 International Diabetes Center, Minneapolis, Minnesota, USA

## Abstract

**Aims:**

In a population at risk for type 2 diabetes (T2DM), we assessed early physical and metabolic markers that predict progression from normal to impaired glucose tolerance (IGT) and T2DM.

**Methods:**

A total of 388 individuals (22% male, age 46 + 11 years) at risk for T2DM were randomized to Standard (n = 182) or Intervention (n = 206) care and evaluated at baseline and 5 annual follow-up visits, including blood pressure, BMI, A1C, lipids, urine albumin/creatinine ratio, VO_2_max, fasting glucose, insulin and C-peptide. The Standard group received results of annual lab tests and quarterly newsletters, while the Intervention group received quarterly newsletters and detailed discussions of lab results, routine self-directed activities, semi-annual group meetings and monthly telephone calls for ongoing support.

**Results:**

Overall, 359 (93%) returned for at least one follow-up visit and 272 (70%) completed the final 5-year assessment. Return rates, changes in measures and incidence of IGT/T2DM were similar between groups. Low cardiorespiratory fitness (VO_2_max) was the most prevalent baseline abnormality. A1C and BMI were significant predictors of IGT/T2DM after controlling for other factors. The risk of IGT/T2DM within 5 years was 17.16 (95% CL: 6.169, 47.736) times greater for those with baseline A1C>=5.8% as compared to those <5.8% (p < 0.0001).

**Conclusion:**

Baseline A1C>=5.8% was a significant predictor of IGT/T2DM within 5 years in a population at high risk for T2DM. A1C is routinely performed among patients with diabetes, however these data and other evidence suggest that it may also be a useful tool for risk assessment and screening.

## Introduction

Diabetes is a chronic disease affecting 171 million of people in the world [1]. The total cost of care for diabetes and its associated complications in 2002 was estimated to be $132 billion [2]. It is estimated that by the time individuals are diagnosed with type 2 diabetes (T2DM), they have had abnormal glucose levels for approximately 10-12 years [3,4]. At the time of diagnosis, as many as 10 to 20% of individuals already has early diabetes complications due to years of undetected hyperglycemia [3,5,6]. Early diagnosis and aggressive treatment aimed at normalizing glycemic levels can minimize the risk of microvascular and macrovascular complications.

Increasing evidence supports the use of interventions that permit changes in diet and physical activity or use of pharmacological treatment to prevent or delay T2DM. These findings provide an impetus for wider implementation of preventive approaches and support the need for effective, early screening of patients at risk. Early markers for diabetes risk may identify individuals who are best to target for interventions aimed at delaying or preventing the onset of clinical disease.

One challenge in diabetes prevention is to identify persons at risk who will benefit from various interventions. It is important to determine what risk factors are associated with the development of impaired glucose tolerance (IGT) and type 2 diabetes in order to identify specific populations for intervention efforts. The current study assessed the efficacy of a community diabetes prevention program on the development of IGT or T2DM in high risk individuals at a relatively early stage of the disease, and evaluated the potential for several markers of diabetes risk to predict the development of IGT or T2DM in individuals at risk.

## Patients and methods

### Study Population

Risk assessment was performed using a tool developed to identify those at risk for T2DM. The specific risk assessment tool used included questions derived from the ADA diabetes screening questionnaire [7] and included family history of diabetes, body weight > 20% over maximum ideal weight, a history of diabetes during pregnancy or having had a baby over 9 pounds, sedentary lifestyle, and additional questions related to the metabolic syndrome (hypertension, dyslipidemia and race/ethnicity). The questionnaire was distributed to the community through the mail, clinics, hospitals and pharmacies, and through local television and public radio by request.

Subjects were eligible for enrollment if they met the following criteria: age 20 to 65 years and family history of diabetes or history of gestational diabetes, or presence of at least one and up to three risk factors for T2DM (obesity, hypertension, dyslipidemia, fasting hyperglycemia) but without all four of these high risk characteristics. Subjects were excluded if they reported having no risk factors, or reported either low activity level or race/ethnicity alone as the only risk factor. In addition, those who were found to have T2DM at baseline were excluded from the trial, as the objective was to target intervention efforts early in the disease process prior to onset of clinical disease.

A total of 1,769 individuals completed the risk assessment questionnaire. Of this group, 1,423 (80%) reported at least one risk factor for type 2 diabetes. Of the 466 who agreed to participate in the study, 418 qualified and completed baseline screening (28 individuals chose to withdraw consent before enrollment, 12 were found to have type 2 diabetes prior to enrollment and 8 had medical conditions that excluded them from participation).

While individuals with IGT were eligible to participate in this study, for the purposes of this report (which considers IGT an endpoint) subjects with baseline IGT were excluded from the analysis. In addition, although enrollment took place prior to the revised ADA criteria for type 2 diabetes [8], our analysis applies the revised diagnostic criteria for the classification of IGT and T2DM at all time points in the study. A total of 388 of the original cohort of 418 were included in the current analysis (30 individuals were excluded due to the presence of IGT or T2DM when the revised ADA criteria were applied retrospectively to baseline measures).

### Randomization

All individuals enrolled in the trial completed baseline laboratory tests and physical assessment, and received written information regarding healthy eating, physical activity and stress management [9]. Subjects were randomized to either the Standard (n = 182) or Intervention (n = 206) group. The Standard group received quarterly newsletters with general information about the importance of healthy eating and physical activity, while the Intervention group received newsletters with more specific information including methods for goal setting, stress management and establishing/maintaining healthy eating patterns, and strategies to increase physical activity. In addition to quarterly newsletters, the Intervention group received personalized interventions based on each individual's readiness to make lifestyle changes. The Intervention group was offered additional semi-annual group education/motivation meetings, self-directed activities, and individual telephone calls each month from trained volunteer dietetic students to offer ongoing support. The goal of the Intervention was to provide participants with information, motivation and support to make healthy eating, physical activity and stress management changes. Subjects in both the Standard and Intervention group received annual laboratory, physical, health risk, psychosocial and nutrition assessments. Results of physical measures and lab tests were discussed in detail with those in the Intervention group, while the Standard group participants received a summary without detailed discussion. In addition, individuals in the Intervention group set personal behavioral goals with the assistance of study staff, while those in the Standard group did not.

### Physical and Laboratory Assessments

The physical assessment evaluated BMI, waist/hip ratio, blood pressure (sitting), and fitness level (as measured by VO_2_max), defined as mL of oxygen consumed per kg body weight per minute (mg/kg/min) during a bicycle ergometer exercise tolerance test (Medgraphics, St. Paul, Minnesota). CVD risk category (Acceptable, Moderate or High Risk) was assigned to each patient based on VO_2_max score using age and gender-specific criteria by Cooper [10] and adapted by Park Nicollet Medical Foundation (Minneapolis, MN) for bicycle testing. All subjects completed fasting plasma glucose (FPG) determination, and those with FPG of 110 mg/dL or greater underwent a 75 g oral glucose tolerance test (OGTT). In addition, A1C (standardized according to DCCT), fasting lipid fractionation (total cholesterol, HDL-cholesterol, triglycerides and calculated LDL-cholesterol), fasting insulin levels, fasting serum C-peptide and albumin/creatinine (A/C) ratio were obtained. The calculation of HOMA-IR was performed using the homeostasis model assessment of Matthews, et al [11].

### Statistical Analysis

Descriptive statistics were calculated for all variables at baseline and at final follow-up visit. Measures are presented as means + SD or percents unless otherwise specified. Measures are presented separately for each disease category: Normal (all FPG values <100 mg/dL), IFG (FPG>=100 mg/dL at any follow-up visit but no IGT/T2DM), and IGT/T2DM (fasting glucose>125 mg/dL or 2 hr OGTT>=140 mg/dL at any visit). FPG of 100 mg/dL or greater were classified as IFG, per the revised ADA criteria for elevated fasting plasma glucose [12]. All subjects who returned for at least one follow-up visit and did not have IGT/T2DM at baseline were included in analyses of follow-up measures. Logistic regression analyses were performed to predict development of IGT/T2DM within 5 years (dichotomous outcome: 1 = IGT/T2DM, 0 = Normoglycemic) using baseline metabolic and physical measures. For inclusion into the regression models, the HOMA = predictor variable was dichotomized as HOMA>2.24 vs <=2.24 (the 75^th ^percentile for baseline HOMA). For the remaining predictors, the most extreme deciles in this study population were used to dichotomize variables for regression models (1 = highest risk decile, 0 = outside the highest risk decile). For VO_2_max, deciles were determined separately for males and females to account for gender differences in oxygen consumption. Variables included in the regression analyses include group (Standard or Intervention), baseline age, gender, A1C, BMI, LDL, HDL, triglycerides, albumin/creatinine ratio and VO_2_max. Metabolic and physical variables that were found to be significant in bivariate regression analyses were evaluated as predictors in the subsequent multivariate analyses.

There were no significant differences between groups with respect to baseline measures, changes over time, or incidence of IGT/T2DM. Logistic regression analysis found that study group was not associated with disease development either alone or in multivariate models. Study group was adjusted for in the analyses and given the similarities between groups (showing no Intervention effect) the current report presents results from a post-hoc analysis for both groups combined in order to determine which risk factors may potentially serve as early markers for IGT/T2DM in this population.

## Results

In this cohort of 388 individuals (22% male, age 46 + 11 years) at risk for type 2 diabetes, a total of 359 (93%) returned for at least one follow-up visit, and 272 (70%) completed the final 5-year assessment. Return rates were similar between the two study groups.

Table 1 lists the prevalence of risk factors in this population at baseline. Low VO_2_max was the most prevalent abnormality, with 83% of the population considered "high risk" based on age and gender-specific criteria (10). Nine percent had baseline A1C values above 5.8%, seven percent had A1C of 6.0% or greater, and more than one-third (36%) had fasting glucose of 100 mg/dL or greater in this population of individuals who were at risk for type 2 diabetes but who did not have IGT or T2DM at baseline. Among those who had FPG<110 mg/dL, 32 people (9%) had A1C>5.8% at baseline, while among those with baseline FPG<100 mg/dL (the revised ADA criteria for normal fasting plasma glucose), 17 people (7%) had A1C>5.8%. Among the 27 people with elevated baseline A1C (>=6.0%), 12 (44%) had FPG>=100 mg/dL and only 2 (7%) had FPG>=110 mg/dL.

**Table 1:** Percent of total study population with high-risk values at baseline (N = 388)

**Measure**	**Value**	**% of Subjects**
FPG (mg/dL)	>=110 >=100	5% 36%

SBP (mmHg)	>=130	36%

DBP (mmHg)	>=85	27%

Cholesterol (mg/dL)	>=200	60%

LDL (mg/dL)	>=130 >=100	48% 82%

HDL (mg/dL)	<40	29%

Triglycerides (mg/dL)	>150	51%

BMI (kg/m^2^)	>=30 >=27	36% 57%

A1C (%)	>=6.0%	7%

Fasting Insulin (μU/mL)	>23	2%

C-peptide (ng/mL)	>4.0	8%

VO_2_max (mL/kg/min)	high-risk score based on age and gender	83%

Table 2 shows the baseline and final values for each variable over the five-year follow-up period among those who returned for at least one follow-up visit for the Standard and Intervention groups combined. Disease status was separated into three categories: 1) the "Normoglycemic" Group includes those with all FPG values < 100 mg/dL, 2) the IFG Group includes those who did not develop IGT/T2DM but whose FPG was 100 mg/dL or greater at any time during the five-year follow-up, and 3) the IGT/T2DM Group includes those who developed IGT or T2DM at any time over the five-year follow-up period. Among the 29 individuals who developed glucose intolerance, 17 developed IGT and an additional 12 developed T2DM over the course of the trial.

**Table 2:** Baseline and final values (mean +SD or %) stratified by 5-year disease incidence among those who completed at least one follow-up visit (n = 359)

	**Normal** **(n = 144)**		**IFG (without IGT/T2DM)** **(n = 186)**		**IGT/T2DM** **(n = 29)**	
	**Baseline**	**Final**	**Baseline**	**Final**	**Baseline**	**Final**

Metabolic						

FPG (mg/dL)	92 + 5	87 + 6	101 + 7	97 + 8	103 + 7	109 + 11

A1C (%)	5.2 + 0.3	5.3 + 0.4	5.4 + 0.4	5.6 + 0.5	5.7 + 0.4	6.1 + 0.5

Insulin (μU/mL)	5.8 + 4.4	5.8 + 3.5	7.7 + 5.2	8.0 + 5.1	11.0 + 7.3	11.4 + 6.8

C-peptide (ng/mL)	2.0 + 1.8	1.8 + 0.7	2.7 + 2.0	2.5 + 1.0	3.6 + 3.2	3.4 + 1.3

HOMA-IR (units)	1.3 + 1.0	1.3 + 0.8	2.0 + 1.4	2.0 + 1.4	2.8 + 1.7	3.1 + 1.9

HDL (mg/dL)	52 + 14	56 + 15	47 + 15	51 + 14	43 + 11	46 + 11

LDL (mg/dL)	123 + 32	122 + 35	130 + 31	123 + 28	143 + 33	129 + 25

Triglyceride (mg/dL)	130 + 70	126 + 70	173 + 128	158 + 98	210 + 93	206 + 92

A/C Ratio (μg/mg)	5.3 + 3.8	8.8 + 9.6	7.5 + 16.6	17.2 + 78.1	7.5 + 16.3	16.3 + 47.4

Physical						

BMI (kg/m2)	26.7 + 5.3	27.7 + 5.8	30.0 + 6.1	30.9 + 6.1	31.0 + 6.5	31.4 + 5.6

SBP (mmHg)	121 + 14	118 + 13	128 + 15	126 + 15	130 + 13	126 + 10

DBP (mmHg)	78 + 8	75 + 8	82 + 8	80 + 8	81 + 9	77 + 7

VO_2_max (ml/kg/min)	22.9 + 5.7	22.7 + 5.8	21.8 + 6.5	22.3 + 7.0	19.4 + 4.6	19.3 + 3.4

Significant predictors of glucose intolerance in bivariate regression analyses included A1C, BMI and VO_2_max. After controlling for the other variables, A1C and BMI remained significant, and VO_2_max was no longer a significant predictor of IGT/T2DM. The final regression model included A1C (p < 0.0001) and BMI (p = 0.0179) as significant predictors of IGT/T2DM within 5 years. Table 3 presents odds ratios, 95% confidence limits and p-values for each variable in the final model, including BMI and A1C (the other variables, including study group, did not influence the odds of IGT/T2DM and were left out of the final model). After controlling for BMI, the odds of developing disease within 5 years were 17.16 (95% CL: 6.169, 47.736) times greater for those with baseline A1C>=5.8% as compared to those with A1C<5.8% (p < 0.0001).

**Table 3:** Association of IGT/T2DM with baseline A1C and BMI (multivariate logistic regression)

	**Unadjusted**		**Adjusted**	
Risk Factor*	**Odds Ratio**	**Odds Ratio**	**95% CL**	**P-value**

A1C>=5.8%	15.763	17.160	6.169, 47.736	<0.0001

BMI>=37.5 kg/m2	3.984	5.133	1.326, 19.879	0.0179

*Values represent the highest deciles for A1C and BMI; all other variables (including study group) were non-significant

## Discussion

The results of our analysis of this study cohort indicated that those individuals with higher baseline A1C values (>= 5.8%) were at significantly higher risk for progression to IGT and type 2 diabetes. While A1C levels have not been routinely recommended as a measure of risk for T2DM nor has A1C level been advocated as a tool to screen for those at high risk, the current observations support the report of Miyazaki et al where elevated levels of A1C in a cohort of Japanese subjects was strongly associated with increased risk of T2DM. Importantly, these authors reported that both A1C and FPG are equally effective as diagnostic tools when compared to 2-h PG [13]. While the authors report an A1C level of 5.7% as the cut-off for maximum sensitivity and specificity level, they also found a significant increase in the prevalence of retinopathy in their study population in those with A1C > 5.8% (tenth decile) [13]. Also, data from the French cohort study, an Epidemiological Study on the Insulin Resistance Syndrome (DESIR), an A1C of 5.9% gave an optimal sensitivity of 64% and specificity of 77% to predict diabetes [14].

It is well established that the risk of diabetes complications increases with increasing A1C among individuals with diabetes. Based on several recent studies, there is increasing evidence that A1C may be associated with adverse health outcomes, including cardiovascular disease [15] and the metabolic syndrome [16], even among those who do not have diagnosed diabetes. Blake, et al, report that among women with no history of diabetes, A1C was higher among those who experienced future cardiovascular events than those who did not, even among those with A1C in the normal range (15). Findings by Osei, et al, suggest that among African American individuals without diabetes whose first-degree relatives have T2DM, A1C between 5.7 and 6.2 (upper tertile) was associated with reduced insulin action and other characteristics of the metabolic syndrome (16). Corpus, et al, reports a higher rate of adverse events among coronary patients without a history of diabetes who had A1C between 6 and 7, compared to those with A1C<6% [17]. Separate reports of Selvin et al [18] evaluating data from the NHANES database and Khaw et al [19] both found that increasing A1C levels within the normal range were associated with a significantly higher risk of cardiovascular disease (CVD). Our findings contribute further evidence supporting that A1C in the upper-normal or near-normal range among those without diabetes is associated with negative health outcomes, including both cardiovascular and metabolic diseases.

The current study demonstrates that low cardiorespiratory fitness (as defined by VO_2_max) is prevalent among individuals at high risk for T2DM (as defined by history of GDM or family history of T2DM, and/or obesity, hypertension and dyslipidemia,).

This study also confirms that high BMI, a risk factor traditionally associated with the metabolic syndrome, is strongly associated with the development of both IGT and T2DM. Furthermore, the trend of increasing A1C in our population seen in the presence of declines in fasting glucose over the course of the study period suggests that postprandial glucose values may be increasing. However this latter observation cannot be confirmed since only subjects with FPG>=110 underwent OGTT in our study.

This community based diabetes risk assessment study demonstrated that A1C value > 5.8% was a significant predictor of the development of IGT or T2DM within 5 years. This study adds further strength to evidence supporting use of integrated measures of glycemia as one tool in identifying those at highest risk for the development of IGT and diabetes. The use of these simple screening measures - if confirmed by other studies - would allow for identification of the higher risk individuals in a community and these individuals could then be targeted for diabetes prevention programs utilizing both lifestyle and medical therapies known to limit the risk of progression to diabetes [20,21]. As Gerstein [22] notes, the increasing prevalence of diabetes indicates that the average A1C level of the general population is likely increasing as well, and because of the reported association between A1C and CVD, a rise in CVD prevalence may occur in the future if large-scale prevention efforts are not implemented. This underscores the importance of public health efforts to modify lifestyle with the goal of preventing T2DM and CVD in populations at risk, and identifying those at risk at an early point when such efforts could potentially prevent the onset or progression of disease.

The community-based strategies included in this study were designed to provide information and support in a cost effective manner. In this population, a set of theory-driven, low cost intervention activities did not result in greater improvements over five years in those who received the intervention compared to those who did not. The reasons for this may be that such an intervention is not as effective as in-person training and counseling sessions. In addition, it is also possible that the semi-annual group meetings and annual lab assessment and review did not occur frequently enough to motivate changes in lifestyle in this population.

In conclusion, the current study provides important information regarding what level of intervention is needed in order to motivate lifestyle changes in a population at risk for T2DM. Further research is needed to determine if an intervention of greater intensity would succeed in impacting lifestyle and metabolic measures, and ultimately prevent or delay T2DM in a population at risk. Importantly, the current study demonstrates that a higher A1C level, even within the normal range, is clearly associated with a substantial increase in the risk of developing clinically significant glucose intolerance within 5 years. Given this and other studies, A1C should be more carefully assessed as a predictor of diabetes in prospective clinical trails and, pending the completion of such studies, should be considered as one measure used by clinicians when screening individuals who are at risk for IGT and type 2 diabetes.

Our study supports increasing evidence of the importance of A1C for risk assessment, screening and diagnostic purposes as indicated recently by the International Expert Committee [23].

## Competing interests

The authors declare that they have no competing interests.

## Authors' contributions

SAOL participated in the collect data, write the manuscript, AAM participated in the collect data and coordination, RLA performed the statistical analysis, DMK helped the statistical analysis, RMB conceived of the study, participated in the design of the study as a senior. All authors read and approved the final manuscript.
